# An Assessment of Survival among Korean Elderly Patients Initiating Dialysis: A National Population-Based Study

**DOI:** 10.1371/journal.pone.0086776

**Published:** 2014-01-22

**Authors:** Shina Lee, Jung-Hwa Ryu, Hyunwook Kim, Kyoung Hoon Kim, Hyeong Sik Ahn, Hoo Jae Hann, Yongjae Cho, Young Mi Park, Seung-Jung Kim, Duk-Hee Kang, Kyu Bok Choi, Dong-Ryeol Ryu

**Affiliations:** 1 Department of Internal Medicine, School of Medicine, Ewha Womans University, Seoul, Korea; 2 Department of Internal Medicine, Wonkwang University College of Medicine Sanbon Hospital, Gunpo, Korea; 3 Department of Public Health, Graduate School, Korea University, Seoul, Korea; 4 Department of Preventive Medicine, College of Medicine, Korea University, Seoul, Korea; 5 Ewha Medical Research Institute, School of Medicine, Ewha Womans University, Seoul, Korea; Department of Medicine and Biomedical Sciences, University of Algarve, Portugal

## Abstract

**Background:**

Although the proportion of the elderly patients with incident end-stage renal disease (ESRD) patients has been increasing in Korea, there has been a lack of information on outcomes of dialysis treatment. This study aimed to assess the survival rate and to elucidate predictors for all-cause mortality among elderly Korean patients initiating dialysis.

**Methods:**

We analyzed 11,301 patients (6,138 men) aged 65 years or older who had initiated dialysis from 2005 to 2008 and had followed up (median, 37.8 months; range, 3–84 months). Baseline demographics, comorbidities and mortality data were obtained using the database from the Health Insurance Review & Assessment Service.

**Results:**

The unadjusted 5-year survival rate was 37.6% for all elderly dialysis patients, and the rate decreased with increasing age categories; 45.9% (65∼69), 37.5% (70∼74), 28.4% (75∼79), 24.1% (80∼84), and 13.7% (≥85 years). The multivariate Cox proportional hazard model revealed that age, sex, dialysis modality, the type of insurance, and comorbidities such as diabetes mellitus, myocardial infarction, congestive heart failure, peripheral vascular disease, cerebrovascular disease, dementia, chronic pulmonary disease, hemiparesis, liver disease, and any malignancy were independent predictors for mortality. In addition, survival rate was significantly higher in patients on hemodialysis compared to patients on peritoneal dialysis during the whole follow-up period in the intention-to-treat analysis.

**Conclusions:**

Survival rate was significantly associated with age, sex, and various comorbidities in Korean elderly patients initiating dialysis. The results of our study can help to provide relevant guidance on the individualization strategy in elderly ESRD patients requiring dialysis.

## Introduction

The incidence of dialysis in elderly patients with end-stage renal disease (ESRD) has been growing throughout the world [1]. The US Renal Data System revealed that the majority of patients initiating dialysis were 65 years or older at the time of their first treatment and also indicated that the rapid growing portion was the population aged over 75 years [2]. However, elderly patients are thought to represent a different proportion across countries as shown in the results from the Dialysis Outcomes and Practice Patterns Study (DOPPS) [3].

According to the 2010 annual report from the Korean nationwide registry program, the incidence and the prevalence of the patients undergoing renal replacement therapy were 181.5 and 1,144.4 per million population, respectively [4], which are less than those reported in epidemiology studies form Taiwan, the United States, or Japan [2]. However, the annual increase in the prevalence has been about 12% during the past decade in Korea [5]. Meanwhile, peak age of patients undergoing dialysis therapy was shifted to older populations, with mean age increasing from 55.2 in 2005 to 58.0 in 2010 [4]. In addition, the percentage of dialysis patients over 65 years had increased to more than 35.0% of overall dialysis population in 2010 [4].

Despite the increasing incidence of elderly ESRD patients initiating dialysis in Korea, there has been a lack of reports regarding outcomes for this population. Moreover, there are many controversies regarding the appropriateness of dialysis initiation in elderly ESRD patients, because age is the most important risk factor for death in the general elderly population and because most elderly ESRD patients have many comorbid conditions affecting mortality, such as dementia, disability, and various cardiovascular diseases [1], [6], [7].

Median survival after dialysis initiation was reported to be only 24.9 months among patients aged 80 or more in the United States [1]. In addition, a recent French study showed that median survival among patients aged 75 years or more had improved from 1.6 years in a 2002–2004 cohort to 2.6 years in a 2005–2007 cohort [8]. However, these studies derived from a Western population. A substantial number of evidences have suggested that there are significant differences in the overall and cardiovascular mortalities in dialysis patients across racial and ethnic groups [9], [10]. To date, however, no studies have been reported on the survival rate and the factors affecting mortality in Asian elderly ESRD patients.

In this study, we evaluated survival rate and elucidated predictors associated with the mortality among elderly Korean patients initiating dialysis.

## Methods

### Ethics Statement

This investigation was conducted according to the principles expressed in the Declaration of Helsinki. The institutional review board at the Korean Health Insurance Review and Assessment Service (HIRA) approved the survey of the study population (No. 3159, 2012).

### Data Source and Study Population

All data used in the study was obtained from the database of Korean Health Insurance Review and Assessment Service (HIRA). In South Korea, all citizens are obliged to join the National Health Security System, which is composed of National Health Insurance and Medical Aid, and is overseen by the Ministry of Health and Welfare.

All medical care expenses for dialysis are reimbursed by HIRA [11], [12]. As such, we were able to identify every ESRD patient in the whole of South Korean population and analyzed the data for all ESRD patients who had started dialysis. We collected data such as unique de-identified number for each patient, age, sex, the type of insurance, list of diagnoses according to the International Classification of Diseases (ICD-10), and kidney transplantation. In addition, the end-point of time to death was confirmed by the Certificate Database (the recorded data of the reasons for changes in eligibility for National Health Insurance or Medical Aid, the death, or emigration) as well as through the National Health Insurance Claims Database.

The comorbidities of each subject were identified by screening the medical history data for the year leading up to the initiation of dialysis therapy. The list of analyzed comorbidities was determined based on suggestions from Charlson et al. [13], and patients were divided into 3 groups [grade 0∼1 (mild comorbidity), 2∼3 (moderate comorbidity), and ≥4 (severe comorbidity)] according to the modified Charlson Comorbidity Index for ESRD patients [14]. ICD-10 codes were used according to the proposed algorithms by Quan et al. [15].

We included data from patients aged 65 years or older who started hemodialysis (HD) or peritoneal dialysis (PD) between January 1, 2005 and December 31, 2008. Patients who survived less than 90 days from the date of dialysis initiation were excluded, and the patients who underwent kidney transplantation or were not deceased until December 31, 2011 were censored.

For the comparison of survival rates between patients on HD and PD, we used both the intention-to-treat (ITT) and as-treated (AT) analyses. The patients were classified according to the treatment modality at the 90^th^ day after commencement of dialysis in ITT analysis, or at the 60^th^ day before death or censoring in the AT analysis.

### Statistical Analysis

Statistical analysis was performed using SPSS software for Windows, version 15.0 (SPSS Inc., Chicago, IL, USA). All data were expressed as mean ± SD or number (%) unless otherwise specified. *P*-values <0.05 were considered statistically significant.

Baseline characteristics of ESRD patients according to age categories were compared using Pearson chi-square test for categorical variables. Kaplan-Meier survival curves were calculated, and the log-rank test was used for the comparison of unadjusted survival rates. In addition, we constructed life tables to estimate the cumulative proportion of survivors at the end of every 1-year-interval during the follow-up period. For delineating predictors for mortality, the Cox proportional hazard analysis was performed. Significant variables in univariate analyses were included for multivariate analysis, and a threshold was 0.10 for retention.

## Results

### Baseline Characteristics

In total, 11,301 patients were included in this study. The number of elderly patients starting dialysis had increased from 2,517 in 2005 to 3,232 in 2008. The demographic characteristics are summarized in Table 1.

**Table 1:** Baseline characteristics.

Age categories	65∼69 (N = 4,491)		70∼74 (N = 3,591)		75∼79 (N = 2,102)		80∼84 (N = 849)		85∼ (N = 268)		Total (N = 11,301)		*P*-value*
Vintage													0.0000
2005	1,101	(24.5)	755	(21.0)	440	(20.9)	176	(20.7)	45	(16.8)	2,517	(22.3)	–
2006	1,055	(23.5)	872	(24.3)	501	(23.8)	200	(23.6)	54	(20.1)	2,682	(23.7)	–
2007	1,115	(24.8)	953	(26.5)	519	(24.7)	209	(24.6)	74	(27.6)	2,870	(25.4)	–
2008	1,220	(27.2)	1,011	(28.2)	642	(30.5)	264	(31.1)	95	(35.4)	3,232	(28.6)	–
Male	2,559	(57.0)	1,977	(55.1)	1,043	(49.6)	419	(49.4)	140	(52.2)	6,138	(54.3)	0.0000
Dialysis modality (ITT)													0.0000
Peritoneal dialysis	976	(21.7)	657	(18.3)	332	(15.8)	122	(14.4)	28	(10.4)	2,115	(18.7)	–
Hemodialysis	3,515	(78.3)	2,934	(81.7)	1,770	(84.2)	727	(85.6)	240	(89.6)	9,186	(81.3)	–
Health security system													0.0499
National HealthInsurance	4,026	(89.6)	3,228	(89.9)	1,872	(89.1)	744	(87.6)	228	(85.1)	10,098	(89.4)	–
Medical Aid	465	(10.4)	363	(10.1)	230	(10.9)	105	(12.4)	40	(14.9)	1,203	(10.6)	–
Comorbidities													
Diabetes mellitus	2,769	(61.7)	2,021	(56.3)	1,031	(49.0)	353	(41.6)	90	(33.6)	6,264	(55.4)	0.0000
Myocardial infarction	230	(5.1)	220	(6.1)	135	(6.4)	44	(5.2)	14	(5.2)	643	(5.7)	0.1587
Congestive heart failure	775	(17.3)	672	(18.7)	463	(22.0)	183	(21.6)	65	(24.3)	2,158	(19.1)	0.0000
Peripheral vasculardisease	342	(7.6)	302	(8.4)	201	(9.6)	72	(8.5)	21	(7.8)	938)	(8.3	0.4549
Cerebrovascular disease	816	(18.2)	674	(18.8)	414	(19.7)	166	(19.6)	57	(21.3)	2,127	(18.8)	0.4549
Dementia	79	(1.8)	102	(2.8)	100	(4.8)	50	(5.9)	20	(7.5)	351	(3.1)	0.0000
Chronic pulmonarydisease	877	(19.5)	810	(22.6)	545	(25.9)	209	(24.6)	70	(26.1)	2,511	(22.2)	0.0000
Connective tissuedisease	141	(3.1)	105	(2.9)	73	(3.5)	29	(3.4)	9	(3.4)	357	(3.2)	0.8180
Peptic ulcer disease	726	(16.2)	637	(17.7)	383	(18.2)	141	(16.6)	41	(15.3)	1,928	(17.1)	0.1699
Hemiparesis	92	(2.0)	77	(2.1)	42	(2.0)	14	(1.6)	4	(1.5)	229	(2.0)	0.8673
Liver disease	424	(9.4)	312	(8.7)	171	(8.1)	65	(7.7)	17	(6.3)	989	(8.8)	0.1405
Any cancer	341	(7.6)	309	(8.6)	195	(9.3)	73	(8.6)	18	(6.7)	936	(8.3)	0.1351
Modified CCI													0.2483
0∼1	1,463	(32.6)	1,142	(31.8)	675	(32.1)	290	(34.2)	90	(33.6)	3,660	(32.4)	–
2∼3	1,589	(35.4)	1,246	(34.7)	722	(34.3)	310	(36.5)	104	(38.8)	3,971	(35.1)	–
≥4	1,439	(32.0)	1,203	(33.5)	705	(33.5)	249	(29.3)	74	(27.6)	3,670	(32.5)	–

* Statistical differences according to age group were calculated in χ^2^ test.

ITT, intention-to-treat; CCI, Charlson comorbidity index.

The mean age was 71.9±5.4 years (range, 65–96 years), and 6,138 (54.3%) were male. The median of follow-up duration was 37.8 months (range of 3–84 months). Patients were divided into 5 categories of age (65∼69, 70∼74, 75∼79, 80∼84, and 85 years or older), with 4,491 (39.7%), 3,591 (31.8%), 2,102 (18.6%), 849 (7.5%), and 268 (2.4%) patients in each group, respectively.

In the ITT analysis, 9,186 (81.3%) and 2,115 (18.7%) were managed with HD or PD at the 90^th^ day after commencement of dialysis, respectively. The number of patients covered by National Health Insurance and Medical Aid was 10,098 (89.4%) and 1,203 (10.6%), respectively. During the follow-up period, 9 patients (0.1%) underwent kidney transplantation.

The number and proportion of patients with comorbid diseases diagnosed before or at the time of dialysis initiation are also described in Table 1.

### The Survival Rates in Korean Elderly Patients Initiating Dialysis

**Figure 1:**
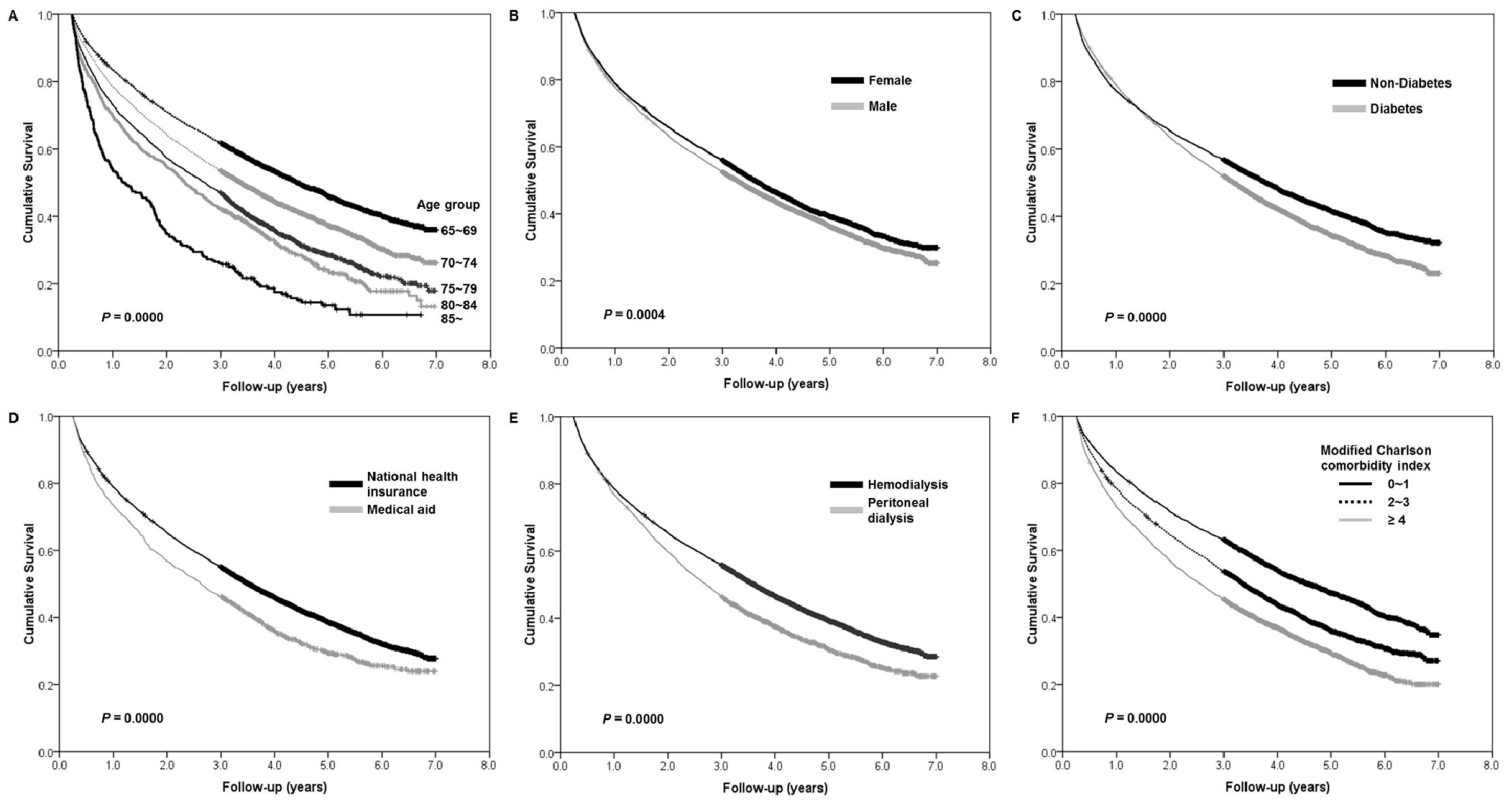
Kaplan-Meier survival curves and comparisons of survival rates by log-rank test. (A) Mortality rates gradually increased with the increment of age categories (*P = *0.0000). (B) Females showed better survival rate compared to males (*P = *0.0004). (C) Survival rate was better for patients without diabetes than that with diabetics (*P = *0.0000). (D) Patients covered by National Health Insurance experienced higher better survival rate compared to Medical Aid beneficiaries (*P = *0.0000). (E) Patients on hemodialysis experienced significant survival benefit over patients on peritoneal dialysis in the intention-to-treat analysis (*P = *0.0000). (F) As the modified Charlson comorbidity index for end-stage renal disease patients increased, survival rates significantly decreased (*P = *0.0000).

Kaplan-Meier survival curves according to age categories, sex, diabetes mellitus, the type of insurance, dialysis modality, and the modified Charlson comorbidity index are shown in Figure 1, which were compared by the log-rank test. The unadjusted survival rates were described in Table 2.

**Table 2:** Cumulative survival rate at each 1-year interval*.

Year	1	2	3	4	5	6	7
All	78.3	64.3	54.1	45.0	37.6	31.6	27.6
Sex							
Male	77.8	63.1	52.5	43.6	36.3	30.0	26.2
Female	78.9	65.8	56.0	46.5	39.2	33.5	29.3
Age groups							
65∼69	83.5	71.2	61.8	53.4	45.9	40.0	35.7
70∼74	78.6	64.3	53.5	44.5	37.5	30.9	26.4
75∼79	73.1	57.3	47.1	35.9	28.4	22.3	18.4
80∼84	70.1	54.7	42.2	33.1	24.1	18.4	15.1
85∼	54.1	35.1	26.1	17.9	13.7	10.7	10.7
Dialysis modality (ITT)							
Hemodialysis	78.6	65.4	55.9	46.7	39.3	33.1	28.9
Peritoneal dialysis	76.9	59.7	46.5	37.5	30.5	25.2	22.1
Dialysis modality (AT)							
Hemodialysis	78.9	65.7	56.3	47.1	39.6	33.5	29.1
Peritoneal dialysis	75.1	57.5	43.6	34.4	28.3	22.8	20.3
Diabetes mellitus							
No	77.4	65.4	56.8	48.3	41.5	35.3	32.2
Yes	79.0	63.4	51.9	42.3	34.4	28.5	23.6
Modified CCI							
0∼1	83.4	71.5	63.2	54.1	47.2	40.6	35.4
2∼3	78.5	64.5	53.7	43.9	36.2	31.0	27.7
≥4	73.0	56.9	45.5	36.9	29.5	22.8	19.0

* All data are presented as percent (%).

ITT, intention-to-treat; AT, as-treated; CCI, Charlson comorbidity index.

The survival rates gradually decreased with increasing age; median survival was 53.1 months for the age group of 65∼69 year, 40.6 months for 70∼74 year, 32.6 months for 75∼79 year, 28.0 months for 80∼84 year, and 14.3 months for the age group over 85 years. The survival rate was decreased with increasing age groups (Figure 1A, *P* *= *0.0000). The unadjusted 5-year survival rate was 37.6% for all elderly dialysis patients, and the rate decreased with increasing age categories; 45.9% (65∼69), 37.5% (70∼74), 28.4% (75∼79), 24.1% (80∼84), and 13.7% (≥85 years) (Table 2).

The survival rate of female patients was significantly higher than that of male (Figure 1B, *P* *= *0.0004). Diabetes significantly affected on the survival outcomes (Figure 1C, *P* *= *0.0000). The type of insurance was also associated with the survival rate, which was higher for National Health Insurance subscribers than for Medical Aid beneficiaries (Figure 1D, *P* *= *0.0000). In the ITT analysis, survival was better for HD patients than for PD patients at all points along the follow-up period (5-year survival rates; 39.3% in the HD patients *vs*. 30.5% in the PD patients) (Figure 1E, *P* *= *0.0000), and the difference in survival rates remained in AT analysis (5-year survival rates; 39.6% in the HD patients *vs*. 28.3% in the PD patients). Furthermore, survival rates were gradually decreased with increasing the modified Charlson comorbidity index (Figure 1F, *P* *= *0.0000). The 5-year survival rate of patients with mild comorbidity (grade 0∼1) was 47.2%, while it was significantly lower at 29.5% for patients with severe comorbidity (grade ≥4) (Table 2).

### Predictors for All-cause Mortality

For the analysis of independent risk factors associated with all-cause mortality, the multivariate Cox proportional hazards analysis was performed with significant variables from univariate analyses (Table 3).

**Table 3:** Results of the Cox proportional hazards analysis for all-cause mortality.

	Univariate			Multivariate*		
	HR	95% CI	*P*-value	HR	95% CI	*P*-value
Age (per 1-yr increase)	1.05	1.04–1.05	0.0000	1.05	1.04–1.05	0.0000
Female (*vs.* Male)	0.92	0.87–0.96	0.0004	0.91	0.87–0.96	0.0002
Medical Aid (*vs.* National Health Insurance)	1.27	1.18–1.37	0.0000	1.25	1.16–1.34	0.0000
Hemodialysis (*vs.* Peritoneal dialysis)†	0.81	0.76–0.85	0.0000	0.75	0.71–0.80	0.0000
Diabetes mellitus	1.17	1.11–1.22	0.0000	1.24	1.18–1.30	0.0000
Myocardial Infarction	1.39	1.26–1.53	0.0000	1.23	1.12–1.36	0.0000
Congestive heart failure	1.30	1.23–1.37	0.0000	1.21	1.14–1.28	0.0000
Peripheral vascular disease	1.19	1.09–1.29	0.0000	1.11	1.02–1.21	0.0142
Cerebrovascular disease	1.42	1.34–1.51	0.0000	1.34	1.26–1.42	0.0000
Dementia	1.64	1.45–1.86	0.0000	1.30	1.15–1.48	0.0000
Chronic pulmonary disease	1.15	1.09–1.21	0.0000	1.09	1.03–1.16	0.0017
Connective tissue disease	1.08	0.95–1.23	0.2571	–	–	–
Peptic ulcer disease	0.97	0.91–1.04	0.4181	–	–	–
Hemiparesis	1.42	1.22–1.65	0.0000	1.20	1.03–1.41	0.0228
Liver disease	1.09	1.00–1.18	0.0401	1.09	1.00–1.18	0.0427
Any malignancy	1.47	1.35–1.59	0.0000	1.48	1.36–1.60	0.0000

* All-cause mortality was adjusted for all parameters with <0.10 of *P*-value in the univariate analysis.

† Mortality rates between patients on hemodialysis and those on peritoneal dialysis were compared in the intention-to-treat analysis.

HR, hazard ratio; CI, confidence interval.

Age, female, Medical Aid, HD as an initial dialysis modality, comorbidities such as diabetes mellitus, myocardial infarction, congestive heart failure, peripheral vascular disease, cerebrovascular disease, dementia, chronic pulmonary disease, hemiparesis, liver disease, and any malignancy were significant independent predictors for mortality.

**Figure 2:**
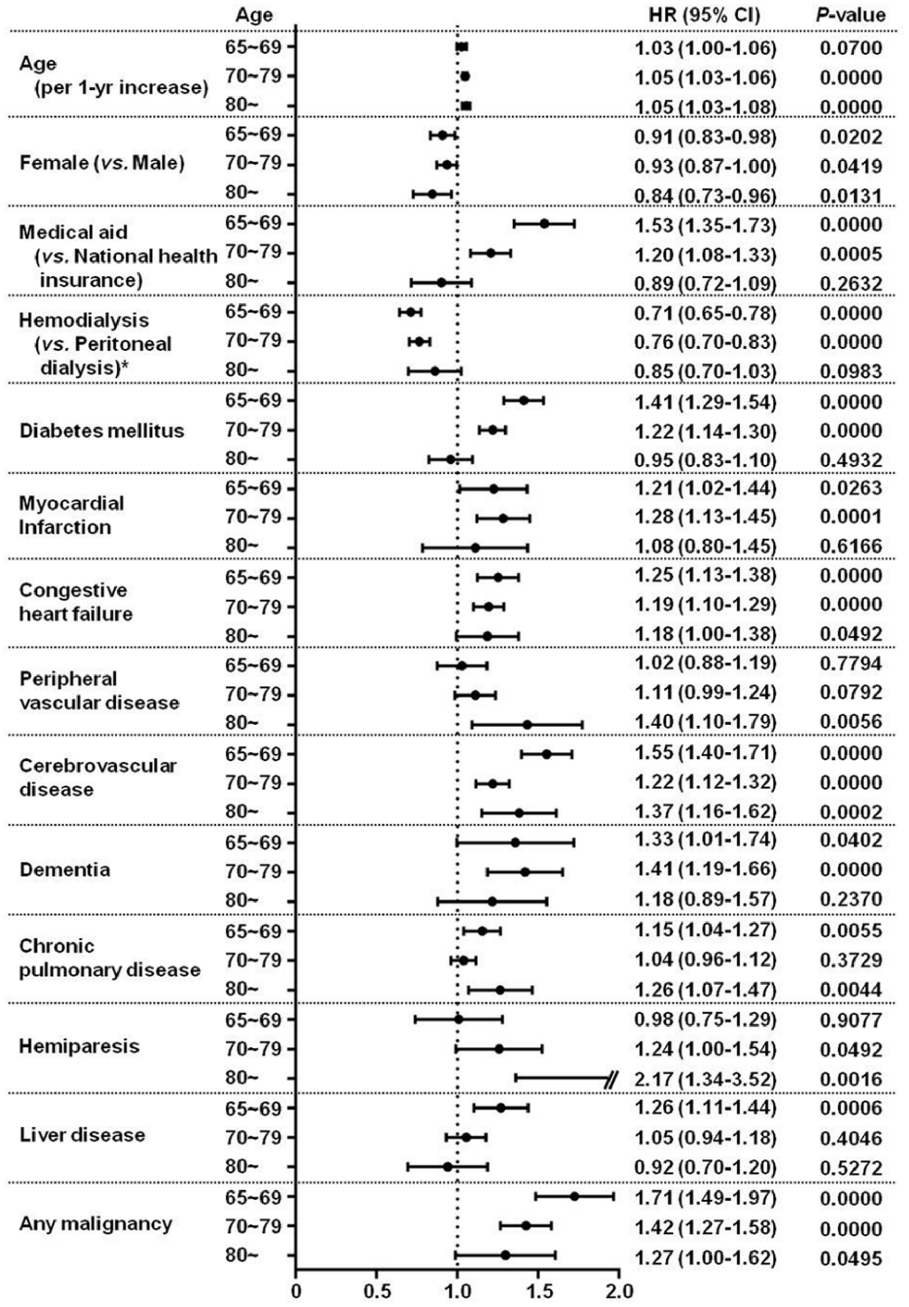
Comparision of hazard ratios of all independent predictors for mortality in the multivariate Cox analysis according to the age categories. Age, peripheral vascular disease, and hemiparesis were the factors by which mortality risk gradually increased with increasing age groups. However, the influences of Medical Aid, peritoneal dialysis, diabetes mellitus, congestive heart failure, liver disease, and any malignancy on the mortality sequentially decreased with aging. * Mortality rates between patients on hemodialysis and those on peritoneal dialysis were compared in the intention-to-treat analysis. HR, hazard ratio; CI, confidence interval.

Furthermore, we compared hazard ratios of all independent predictors for mortality in the multivariate Cox analysis according to the age categories (Figure 2). For the simplicity in comparison, patients were divided into 3 categories of age (65∼69, 70∼79, and 80 years or older), with 4,491 (39.7%), 5,693 (50.4%), and 1,117 (9.9%) patients in each group, respectively. Age, peripheral vascular disease, and hemiparesis were the factors by which mortality risk gradually increased with increasing age groups. However, the influences of Medical Aid, peritoneal dialysis, diabetes mellitus, congestive heart failure, liver disease, and any malignancy on the mortality sequentially decreased with aging.

## Discussion

In this study, we evaluated survival rates among elderly Korean ESRD patients initiating dialysis. More than 60% of elderly patients had survived for 2 years after initiation of dialysis. Even among the very elderly patients, the median survival was 28.0 months for patients 80∼84 years of age; 14.3 months for patients 85∼89 years of age; and 13.2 months for patients 90 years of age or older, respectively. The elderly dialysis patients in this study experienced a lower death rate when compared to that reported in a previous study of US octogenarians and nonagenarians starting dialysis, in which median survival rate was 15.6 months for patients aged 80∼84 year; 11.6 months for patients aged 85∼89 year; and 8.4 months for patients aged 90 year or older [1]. Several studies have also reported that median survival of the patients older than 75 years was around 2 years after the first dialysis [16], [17], [18]. In addition, a UK study of dialysis patients aged 70 or older found that overall 1-yr survival was 71% [19], which is slightly different from the results of this study (74.8% of oveall survival rate at 1 year among patients aged 70 or older).

There has been a controversy regarding initiation of dialysis in very elderly patients. Although a randomized controlled study is the most reliable and informative method of comparing outcomes between conservative care and dialysis initiation, such a trial is difficult to perform due to ethical concerns. When standardized mortality ratios were calculated in comparison with the general population, the ratio for the dialysis patients decreased from 26.7 in the 18- to 44-year-old group to 3.5 in the ≥85-year-old group, which implicated that older ESRD patients experienced less excess mortality, and age *per se* is more important factors for mortality in the elderly than in the younger age group [6]. Moreover, elderly patients have various chronic medical diseases concurrently, which have profound effects on their survival [20]. Therefore, very elderly patients may not always be considered as suitable candidates for dialysis. In this study, age and various comorbidities were the main determinants for mortality, which is consistent with previous reports. However, we suggest that the benefit and risk of dialysis initiation should be weighed in each patient because survival rate for elderly ESRD patients is not dismal in Korea and because the individualization strategy using predictors for mortality is feasible.

Although life expectancy is usually greater for females than for males in the general population, sex has not been considered as a risk factor for death in ESRD patients [4], [6], [19]. In this study, however, the survival rate of female was higher than that of male in Kaplan-Meier analysis, and female still conferred a relative survival benefit over male after adjustment for baseline variables. Because there was no distinct reason for the difference across the sex, we suggest that racial/ethnic difference could be taken into account.

In a previous report, diabetes was not an independent predictor for mortality in an older patient group [19], while it was a significant risk factor for mortality in the present study. In addition, myocardial infarction, cerebrovascular disease, or chronic obstructive pulmonary disease were also independent risk factors for mortality in our study. Diabetes could take more time to influence mortality rates compared to concomitant comorbidities such as various vascular diseases. Thus, aging *per se* may offset the effect of diabetes on mortality in the very elderly patients. In this study, patients between 65 and 69 accounted for 39.7% of the total participants. Therefore, it was assumed that these comorbidities still had a significant effect on mortality because life expectancy was long enough to be affected.

The National Health Security System of South Korea consists of National Health Insurance and Medical Aid, which respectively provide healthcare coverage for 96.3% and 3.7% of the whole population in 2006 [21]. The Medical Aid Program was established for low-income households, thus it roughly represented lower socioeconomic status in this study. Several studies have proved that low socioeconomic status was related with poor outcomes in patients with chronic kidney disease as well as patients on HD [22], [23]. In accordance with these reports, mortality rate was significantly worse for the Medical Aid beneficiaries than for patients covered by National Health Insurance. This difference between insurance remained as a strong predictor for mortality even after adjustment for baseline covariates.

Although the debates continue on the choice of dialysis modality in elderly patients, HD is preferred over PD in many countries. In our study, the use of HD was about 5 times greater than PD. HD patients experienced higher survival rate than PD patients during the follow-up period, and multivariate analysis revealed that the initial choice of HD had 25% survival benefit over PD. Previous studies have revealed that HD provided better survival outcomes than PD in elderly patients, especially after 180 days of dialysis initiation [23], [24], [25], [26]. In addition, the registry data from Australia and New Zealand suggested that PD may be advantageous initially in younger patients (<60 years) without comorbidity [22]. The Dutch registry data noted survival advantage for PD in young non-diabetic patients [27]. These findings require more judicious approach in considering PD as an initial dialysis modality in elderly patients.

In this study, we accepted the chronological age of 65 years as a definition of ‘the elderly’. However, the patients aged 65 years or older were thought to be wide-ranged and were likely to have different characteristics. In accordance with this assumption, there were significant differences in the independent predictors according to the age categories. Age *per se* and the factors affecting the degree of disability such as peripheral vascular disease or hemiparesis were more significant with increasing age groups. However, the influences of traditional risk factors such as socioeconomic status, dialysis modality, and comorbidities such as diabetes mellitus, congestive heart failure, liver disease, or any malignancy on the mortality sequentially decreased with aging.

As with other registry-based studies, this study also has limitations inherent to such study design. First, potential confounding factors for mortality were unavailable, such as data regarding residual renal function, critical laboratory results, biomarkers of inflammation or nutrition, and dialysis doses. Second, we could not gather the cause of death for each individual. Finally, quality of life is as an important outcome variable as mortality rates in elderly patients, but this could not be evaluated in this study.

In spite of these limitations, our study has provided several clinically relevant points. Although there have been many reports on outcome in elderly ESRD patients, the findings from these studies could not be generalized to the populations outside of Western countries. Due to the large sample size that includes the entire population of dialysis patients with relatively long follow-up (up to 84 months) periods of this study, the results may be extrapolated to the current status of dialysis outcome in elderly Asian populations, albeit differences in policies or clinical practices do exist across the countries. In addition, Korean elderly ESRD patients were found to have distinctive predictors for mortality, such as sex, diabetes mellitus, and various comorbidities, which should be considered during decision-making for dialysis initiation.

Taken together, the findings suggest that survival outcomes in elderly patients initiating dialysis are different from those of previous reports, probably due to racial and ethnic differences. The results can help to provide relevant guidance on the individualization strategy in Asian elderly ESRD patients.
